# *Education:* The role of the first siderophore cephalosporin Fetcroja^®^ (cefiderocol) in UK clinical practice: introduction

**DOI:** 10.1093/jacamr/dlab051

**Published:** 2021-06-15

**Authors:** Abid Hussain

**Affiliations:** Public Health England Public Health Laboratory, Birmingham B9 5SS, UK

## Abstract

Welcome to this special *JAC-Antimicrobial Resistance* Supplement, which is sponsored by Shionogi BV. The march of increasing Gram-negative resistance is relentless, and in the last 10 years we have seen a development in the pipeline of antimicrobials targeting the most resistant Gram-negative bacteria. Within this Supplement, we will present the current landscape for Gram-negative treatments, the launch data for cefiderocol, UK potential positioning and examples of early use. **The prescribing information for cefiderocol is available at: https://shionogi-eu-content.com/gb/fetcroja/pi.**

In England, the incidence of antibiotic-resistant bloodstream infections increased by 32% from 2015 to 2019, most markedly in Enterobacterales.^1^ This in part was due to selection pressure through antimicrobial use, with a switch from piperacillin/tazobactam to third generation cephalosporins in 2017, and then the subsequent consumption levelling off. Many laboratories have moved to local confirmation of the mechanisms of carbapenem resistance in Enterobacterales, and carbapenem MICs for some carbapenemase producers may be below clinical breakpoints^2^ with resistance due to ESBL or AmpC expression coupled with porin loss. This laboratory data should be interpreted in a clinical context before a decision around prescribing antimicrobials is made. Antimicrobial resistance increased modestly in *Pseudomonas* spp. and *Acinetobacter* spp. between 2015 and 2019 by 2%. 

The evolution of the COVID-19 pandemic had a detrimental effect on antimicrobial stewardship, with an increase in total antibiotic prescribing DDDs per 1000 admissions in Quarter 1 of 2020–21.^3^ A lack of data describing the evolution of the disease coupled with concerns around bacterial coinfection may have driven this, although further robust stewardship is required to bring the prescribing of certain antimicrobials, such as quinolones, under control. Thankfully, total consumption across NHS Trusts in England has returned to pre-COVID levels in Quarter 2. These concepts are explored in Professor Livermore’s manuscript examining the future of antimicrobial resistance.^4^

Amongst the new agents that have been approved in Europe is cefiderocol, a novel siderophore cephalosporin, which is licensed for the treatment of infections due to aerobic Gram-negative bacilli in adults with limited treatment options.^5^

The structure of cefiderocol consists of a cephalosporin backbone with a catechol moiety at the 3-position side chain. Bactericidal activity is conferred by the cephalosporin core, which binds primarily to penicillin-binding proteins, inhibiting peptidoglycan cell wall biosynthesis. The catechol moiety differentiates cefiderocol from other cephalosporins as it chelates ferric (Fe-III) iron to mimic natural siderophores, allowing cefiderocol to be actively transported into the cell. This process increases the periplasmic concentration of cefiderocol by circumventing non-specific resistance due to porin loss or efflux. In this way, cefiderocol’s bactericidal activity is enhanced relative to carbapenems, other cephalosporins, and β-lactam/β-lactamase inhibitor combinations.^6–8^

The utilization of cefiderocol in clinical practice is determined not only by *in vitro* susceptibility testing but also by the breadth of the licensing. In the case of the former, EUCAST breakpoints are available.^9^ In the case of the latter, Dr Karas and Professor Edgeworth^10^ review the relevant clinical data from the licensing data and share some reflections on how this agent would be used in clinical practice, illustrated by a case of compassionate use.

Whilst framework recommendations for carbapenemase-producing Enterobacterales were published last year, the most complex and challenging cases can be as a result of infections with MDR non-lactose fermenting organisms. To illustrate this point, Drs Chavda and Gilchrist^11^ highlight the clinical utility of cefiderocol in managing osteomyelitis, whilst Dr Mabayoje and colleagues^12^ describe the utility of cefiderocol in a patient with prosthetic joint infections. Professor Falcone^13^ shares his experience of using cefiderocol as a rescue therapy in the ICU.

*In vitro* studies have demonstrated that cefiderocol has excellent activity against Gram-negative bacilli, both Enterobacterales and non-lactose fermenters such as *Pseudomonas aeruginosa, Acinetobacter baumannii, Burkholderia cepacia* and *Stenotrophomonas maltophilia*. The increasing reports of successful treatment of complex infections, particularly those that are deep seated and are associated with prosthetic devices, is particularly encouraging. Cefiderocol use should be tightly managed with an antimicrobial stewardship programme but can be seen as a sensible alternative to some of the nephrotoxic agents in established use. The lack of requirement for therapeutic drug monitoring and favourable adverse event profile allows prescribers more flexibility when targeting therapy if the resistance mechanisms are known.

## Transparency declarations

This article is part of a promotional Supplement developed and sponsored by Shionogi B.V. The accompanying video formed part of an educational webinar for which the author received a speaking honorarium. The material underwent peer review by the Supplement Editors. Editorial assistance to Shionogi Europe was provided by Page Medical.
